# A Brain-Computer-Interface for the Detection and Modulation of Gamma Band Activity

**DOI:** 10.3390/brainsci3041569

**Published:** 2013-11-18

**Authors:** Neda Salari, Michael Rose

**Affiliations:** NeuroImage Nord, Department of Systems Neuroscience, University Medical Center Hamburg Eppendorf, Martinistrasse 52, D-20246 Hamburg, Germany; E-Mail: n.salari@uke.de

**Keywords:** brain-computer-interface, neurofeedback, gamma oscillations

## Abstract

Gamma band oscillations in the human brain (around 40 Hz) play a functional role in information processing, and a real-time assessment of gamma band activity could be used to evaluate the functional relevance more directly. Therefore, we developed a source based Brain-Computer-Interface (BCI) with an online detection of gamma band activity in a selective brain region in the visual cortex. The BCI incorporates modules for online detection of various artifacts (including microsaccades) and the artifacts were continuously fed back to the volunteer. We examined the efficiency of the source-based BCI for Neurofeedback training of gamma- and alpha-band (8–12 Hz) oscillations and compared the specificity for the spatial and frequency domain. Our results demonstrated that volunteers learned to selectively switch between modulating alpha- or gamma-band oscillations and benefited from online artifact information. The analyses revealed a high level of accuracy with respect to frequency and topography for the gamma-band modulations. Thus, the developed BCI can be used to manipulate the fast oscillatory activity with a high level of specificity. These selective modulations can be used to assess the relevance of fast neural oscillations for information processing in a more direct way, *i.e.*, by the adaptive presentation of stimuli within well-described brain states.

## 1. Introduction

Brain-Computer-Interface (BCI) can be used as a non-invasive method to enhance the human ability to regulate electrical brain activity. BCI applications are applied as active and/or reactive BCI. With electroencephalography (EEG)-based active BCI (Neurofeedback), brain signals are recorded from the scalp and relevant components are extracted in near real-time and fed back to the individual, *i.e.*, in the form of visual information. The individual uses the feedback information in order to learn how to deliberately modify a particular brain activity. Reactive BCIs can be used to trigger specific commands as specific frequencies are classified, such as the movement of a robotic arm or the presentation of visual stimuli.

Neurofeedback methods are used to train different neurophysiologic signals at different cortical areas. Several studies have used changes in evoked potentials as a feedback signal [1,2,3], resulting from perception and processing of stimuli. Further neurofeedback methods are based on neuronal oscillations occurring at different frequency ranges in different brain areas. Mu (8–13 Hz) and/or central beta (13–25 Hz) rhythms, for example, are recorded at the sensorimotor cortex and are related to motor preparation and imagination of movements [4,5]. Slow cortical potentials (1–2 Hz) have been exploited as a source of control to train patients with severe motor disabilities, such as ALS (Amyotrophic Lateral Sclerosis), to control a spelling device in order to communicate [6]. Furthermore, neurofeedback has been applied for the treatment of attention deficit disorder [7,8] or epilepsy [9]. Apart from the clinical application of neurofeedback, this method offers the opportunity to modulate activity in different frequency bands and topographic areas, and to examine a more direct relation between oscillatory brain states and behavior.

Oscillations in the gamma band (around 40 Hz) play a functional role in many aspects of information processing [10,11,12,13]. A recent study has revealed that also prestimulus gamma-band fluctuations in lateral occipital areas correlate with certain aspects of visual processing [14]. However, correlative findings cannot establish a causal link between the observed oscillations and distinct steps of information processing. Several methods like optogenetic techniques [15], TMS (Transcranial Magnetic Stimulation) [16], or direct electrical stimulation [17] are used for selective modulations of neural activity to establish more causal relations between brain regions, oscillatory activity, and functions. In line with these methodological approaches BCIs can be used for an online manipulation of ongoing oscillations and would allow a more direct analysis of the functional relevance of these higher frequencies. Furthermore, an EEG based BCI allow a complete non-invasive method for a modulation of ongoing oscillatory activity. Although the gamma band is highly important and plays a functional role in cognitive processing, it is a rare explored frequency range in neurofeedback experiments. Recently, it was demonstrated that the use of gamma-band activity can improve the performance of BCI systems [18,19,20]. In a preliminary study we showed that gamma-band activity can be modulated by neurofeedback training, and that this approach can be used to address the functional role of gamma-band oscillations. Using a reactive BCI we presented visual stimuli online during increased gamma band activity over the visual cortex and demonstrated a direct influence of prestimulus gamma-band activity for visual object processing [21].

Several important aspects have to be considered during the development of a Brain-Computer-Interface based on gamma band oscillations. Oscillations in the gamma band are extremely susceptible to artifacts, such as eye or muscle movement, which occur in a common frequency range. In particular, microsaccadic eye movements can affect gamma band activity [22]. Therefore, our BCI has to estimate all possible artifacts and inform volunteers on confounding activity to assure that the volunteers learn to modulate the neural activity and to avoid confounding muscle or eye activity. This should support the volunteer to develop an artifact-free strategy for the enhancement of the neural activity and result in a more effective training.

The visual display of the neural feedback signal should include all relevant information in a compact form to avoid distraction, allow a rapid extraction of the information, and, at the same time, motivate participants to learn to increase gamma-band activity.

Distinct information processing steps are realized by oscillatory activity with distinct frequency characteristics in circumscribed neural areas. For instance, gamma-band oscillations in the lateral occipital cortex (LOC) are related to visual object processing [23]. In contrast, it was shown that alpha oscillations in occipital parietal areas impair visual processing [16]. To disentangle the functional relations of the different oscillations, a selective modulation of both bands using neurofeedback could be used.

Oscillations in the alpha band (8–12 Hz) represent a resting state and have been explored and successfully trained in a variety of neurofeedback experiments [24,25,26]. In the present study, both alpha- and gamma-band activity, in a circumscribed region of the visual cortex, are used as a feedback signal in an alternating sequence to train the volunteers to switch between an increase of gamma- or alpha-band activity. We tested, whether the volunteers could learn to selectively increase alpha- and gamma-band activity, and evaluated the specificity of this approach in the frequency and spatial domain. In particular, we aimed to examine whether modulation of these frequencies was limited to the trained frequency bands and to the trained brain region.

Gamma-band oscillations are important for visual object processing and a functionally well-defined neural area for this process is the LOC [27,28]. It has been demonstrated that combining low-resolution electromagnetic tomography (LORETA) with the neurofeedback technique more spatially specific information can be derived [29]. Therefore, in the present study neural activity of the alpha and gamma band was estimated online from a region of interest (ROI) that covers bilateral LOC. In an alternating sequence volunteers were either instructed to increase alpha-band activity or to enhance gamma-band activity.

With our advanced BCI method we aimed to investigate following research questions: (1) Does an online feedback of eye and muscle artifacts in addition to the feedback value improve BCI training? (2) Can the volunteers learn to switch between modulating two different frequency bands? (3) Can we use source information to train alpha- and gamma-band frequencies in a defined region? (4) Which areas are affected topographically by alpha- and gamma-band training, and are they restricted to the selected LOC? (5) Can a selective modulation of visual gamma-band activity be achieved by a non-invasive BCI?

## 2. Materials and Methods

### 2.1. Volunteers

Eight healthy, right-handed volunteers with normal or corrected to normal vision participated in the experiment (mean age 25, five male, three female). All participants had no prior BCI experience. The experiment was approved by the ethics committee, and volunteers gave written informed consent prior to the experiment.

### 2.2. BCI Method

#### 2.2.1. Technical Setup

The volunteer sits in a room and watches a liquid crystal display monitor with a viewing distance of 1 m. EEG was measured from 58 active electrodes at standard locations (ActiCap, Brain Products, Gilching, Germany) at sample rate of 250 Hz and all channels were referred to Cz. In addition, we recorded vertical and horizontal EOG (electrooculogram) from above *versus* below the left eye (supraorbital VEOGS and infraorbital VEOGI) and from the outer canthi of the eyes (left HEOGL, right HEOGR), for detecting eye movements. Neck muscle activity was derived bipolar about 20 cm below the occipital electrodes over the trapezius muscle and electrode impedance was kept below 10 kOhm.

The BCI method was realized with two connected computers. The first PC (personal computer) receives the analogous voltage change of the measured channels and stores the data in raw format into the database for later offline analyses. Additionally, it acts as a remote data access (RDA) server, which allows the EEG data to be passed via TCP/IP (transmission control protocol/internet protocol) to other computers in a network. In this process, a second computer runs a corresponding client (RecView, Brain Products, Gilching, Germany), which receives data over the Ethernet network for real-time data analysis and delivers the appropriate visual feedback to the participant. The RecView software was mainly used to receive online data and for the usage of the LORETA module.

#### 2.2.2. Online Data Processing

The source based BCI experiment with artifact control was designed to train individuals to increase their current density power in the alpha- (8–12 Hz) or gamma- (around 40 Hz) frequency band in the LOC. In order to estimate the current density power of alpha and gamma frequencies in the LOC and the use for the BCI, the recording EEG signals were processed online with the following steps: (1) Preprocessing; (2) LORETA transformation; (3) Feature of interest extraction; and (4) Visually presentation to the participant.

**Figure 1:**
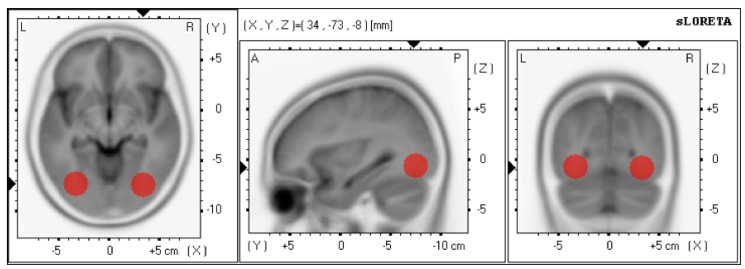
Selected ROIs in the right and left LOC for neurofeedback training.

**Figure 2:**
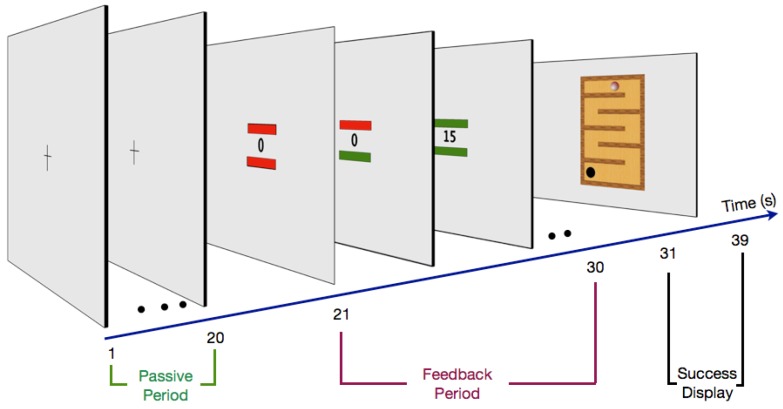
The neurofeedback design. Neurofeedback training: during the passive period (20 s) volunteers fixated the central cross. This period was used to assess an actual baseline value of alpha or gamma current density power in the ROIs. In the feedback period (second 21–30) participants tried to increase the current density power in the ROIs (value at fixation) and at the same time avoid EOG (bar above value) and EMG (electromyography) (bar below value) artifacts by keeping the bars *green*. As an artifact occurred (at least one of the bars *red*) the presented value was set to zero. The success of the intentionally increased artifact free gamma or alpha values was presented after the feedback period (success display).

(1) The custom written preprocessing module included the bipolar calculation for eye and muscle channels and the removal of amplitude drifts in EOG channels, in order to prepare selective channels for artifact detection (see Section 2.2.4. Artifact Detection Filters). After the preprocessing of incoming data and artifact detection, we applied a Butterworth filter on all channels (except for EMG and REOG channel) around 40 Hz (30–45 Hz, slope 48-db per octave, order 8) to extract gamma-band activity during gamma sessions. For the alpha sessions the filter was set around 10 Hz (8–12 Hz, slope 48-db per octave, order 8) to extract alpha-band activity.

(2) In order to train gamma- or alpha-band oscillation in the LOC, the filtered signals were then transmitted to the LORETA module from the RecView software (see Section 2.2.4 Online Source Localization). Using information acquired from electrodes placed on the scalp, the LORETA method estimates the distribution of electrical neural activity in three-dimensional space [30]. In particular, we defined the regions of interest in the left LOC [(*x*, *y*, *z*) = 34, −73, −8] and right LOC [(*x*, *y*, *z*) = −34, −73, −8] (Sphere 12 mm, encompassing 7.2 cm^3^ each ROI) (Figure 1), based on previous EEG and fMRT studies [28]. Hence, the LORETA module estimates the average current density amplitude in the defined ROIs and derives a new EEG channel for each ROI (left LOC, right LOC).

(3) In the custom written feature extraction module both the estimated activity in the LOC for gamma- or alpha-band activity and the detected artifacts were calculated (see Section 2.3 Procedure).

(4) The change of gamma- or alpha-band activity in the LOC was visualized by a value at fixation. In addition, two bars were added above and below the feedback value, representing EOG and EMG artifacts (see Section 2.3 Procedure and Figure 2).

#### 2.2.3. Online Source Localization

LORETA is an inverse solution technique, which was used to estimate real-time current density in the LOC for alpha or gamma band oscillations. The virtual MR (magnetic resonance) anatomical images are made available by the Montreal Neurological Institute of McGill University (Montréal, Canada).

The LORETA method [30] deals with the EEG inverse problem stated as N electrode scalp measurements at time *t*, estimate the source current density within a three-dimensional solution space generating them. As the number of sources is greater than the scalp measurements, there are an infinite number of sources that can explain the measured electrical potential difference on the scalp.

If the source within a 3D solution is known, the electrical potentials on the scalp can be determined with a unique solution, known as the forward solution [30]. LORETA applies a realistic head model to calculate the distribution of electrical potentials for given source locations (three-shell head model to the Talaraich human brain atlas. available as a digital MR Image from the Brain Imaging Centre, Montreal Neurological Institute). For more detailed information on the LORETA method, please refer to [30].

#### 2.2.4. Artifact Detection Filters

In order to assure the EEG measurement of artifact free brain signals three artifact detection filters were implemented. The filters detected for electrical activity generated by muscle contraction in jaw, neck or shoulders (EMG) and activity generated by eye blinks, movements (EOG), or microsaccades.

An overview of detected artifact types is given in Table 1.

**Table 1:** Overview of detected artifact types.

Artifacts	Type	Detection	Channels	Feedback information
Eye artifacts	Eye blinks	Signal threshold	VEOG	Integrated in upper bar (feedback period)
	Horizontal eye movement	Signal threshold	HEOG	Integrated in upper bar (feedback period)
	Vertical eye movement	Signal threshold	VEOG	Integrated in upper bar (feedback period)
	Microsaccades	Signal threshold	REOG	Success display
Gamma band specific artifacts	Neural source of gamma activity	Variable threshold = gamma band activity in ROI channels	VEOG HEOG	Integrated in upper bar (feedback period)
Muscle artifacts	Neck muscle activity	Threshold = mean 70–80 Hz activity during passive baseline	N	Integrated in below bar (feedback period)
	Jaw clenching	Threshold = mean 70–80 Hz activity during passive baseline	T7 T8	Integrated in below bar (feedback period)

##### 2.2.4.1. EOG

For the detection of eye blinks, a bipolar vertical EOG (VEOG) channel was calculated online as the difference between VEOGS and VEOGL and the resulting channel was high-pass filtered (0.5 Hz) to remove dc-offsets. In a pre-study with twenty participants we derived thresholds for blink detection and eye movement detection. The average value of detected blinks over all volunteers was 140 μV with a standard deviation of 104 μV. Thus, the threshold was set at 50 μV to assure the detection of smaller blinks. Furthermore, blink artifacts are large amplitude distortions followed by negative voltage deflections. The negative deflections usually appear within a range of 300 ms around the positive peak. This information was used to improve the detection of blinks and large vertical eye movements. Therefore, as the VEOG signal exceeds the threshold value, the maximum and minimum values are identified and if the difference between the peaks exceeds 60 μV, then a blink is detected.

For the detection of eye movement, a bipolar horizontal EOG (HEOG) channel was calculated as the difference between HEOGL and HEOGR. A high-pass filter was applied on the bipolar derived electrode HEOG (0.5 Hz) to reject dc offset. Vertical horizontal and round eye movements produce square shaped EOG [31], while eye blinks produce spikes. Hence, as a value exceeded the threshold value (average amplitude = 64 μV, sd = 34 μV, threshold = 20 μV), we furthermore specified that all following data values within 40 ms had to be greater than the threshold value.

In order to assure that the neural source of gamma-band increase originates from the LOC and not from the EOG channels, we added a further condition specified for the gamma-band sessions. If the percent change of gamma-band activity in the EOG channels exceeded the gamma-band change in the ROIs, then the upper bar turned red and the feedback value was set to zero. By addressing this consideration, we assured that the maximum increase of gamma band activity was generated from the ROIs.

Thus, anytime a blink, an eye movement or higher gamma activity in the EOG channels as compared to the ROI channels occurred, the volunteer was visually informed as the bar above the presented gamma value turned red. Respectively, the EOG bar turned green if no EOG artifacts occurred within the passed 1000 ms.

As shown in Table 1, the upper bar represents different sources of eye artifacts. The meaning of the different types of the online feedback was explained to the volunteers during the introduction at the beginning of the experiment. After each training session, an artifact detection overview with a summary of detected artifacts was presented to the volunteers. Thus, after each session the volunteer was informed, whether the eye artifacts were due to eye blinks, eye movement, microsaccades, or gamma-band specific artifacts.

##### 2.2.4.2. Microsaccades

A general concern has aroused towards the neural origin of gamma band activity in non-invasive recordings. A recent study has proposed that scalp recorded gamma-band oscillations in parietal electrodes in EEG-data are influenced by microsaccades instead of neuronal processes [22]. In order to assure the neural origin of the measured gamma band increase and to estimate the influence of saccadic activity, we applied a recently proposed saccadic spike potential (SP) detection method [32], which allows an accurate detection of microsaccades directly in EEG traces without acquiring fast eye-tracking.

Ocular artifacts such as the SP are most prominent in the peri-orbital electrodes when referenced to occipital or parietal electrodes [33]. Thus, for the offline detection of SPs, a further “radial” electro-oculogram channel (REOG) was derived as recommended [32]. The REOG channel is defined as the average of all EOG channels referenced to Pz:

REOG = (HEOGR + HEOGL + VEOGS + VEOGI)/4 − Pz
(1)

As suggested in previous experiments the REOG channel was filtered with a Butterworth IIR filter (BPF) of an order of 6, with a pass-band of 30–100 Hz for the detection of microsaccades.

The detection threshold was set at 2 standard deviations from the mean of the filtered signal. As the filtered signal was computed online we applied an online “running” standard deviation to avoid memory access [34,35]. Thus, the standard deviation is refreshed with each incoming online data from the passive and feedback periods. The success display period was not encountered for the calculation of the standard deviation as the period served as a break. After a short initialization phase within the passive period the standard deviation tends to a stable value.

We exploited the saccade detection algorithm to determine the amount and mean amplitude of detected SPs in both passive and feedback periods to test for saccadic changes between the periods and across training. The REOG trace yields reasonable accuracy for saccades above 0.2, which should be sufficient to detect saccadic activity in visual paradigms [32]. During the success display volunteers were informed about their average SPs per second and SP amplitude in the two passive periods and in the passed feedback period. Thus, volunteers are informed if they exceed the average SP amount or amplitude in the passed feedback period.

##### 2.2.4.3. EMG

Most common sources of EMG are muscles, when closing, opening, or clenching the jaw. These muscle contractions generate high gamma frequencies, which are measurable close to the temporal locations (T7, T8). Moreover, muscle contraction in the neck can generate high frequencies as well. To control for possible EMG contamination, channels T7, T8 and the bipolar derived neck channel (N) were subjected to sixth order Butterworth filtering in the bandpass 70–80 Hz. During the passive periods the average activity in the channels T7, T8, and the neck was calculated and set as a baseline for the following feedback periods. Thus, if the percentage change of 70–80 Hz activity in the EMG channels was higher than in the ROIs, then the bar below the display value turned red.

### 2.3. Procedure

Participants were trained for an hour, once a week, over a period of three weeks. Each training day consisted of eight sessions of gamma-band training and eight sessions of alpha-band training, which were presented in an alternating sequence. A session started with a passive period (20 s) followed by eight feedback periods (10 s). The design of the experiment was clearly arranged with a simple cross during the passive periods and a feedback value with two artifact bars during the feedback periods. The bars were placed central and close to the feedback value, in order to keep the volunteer focused to the feedback value and to avoid eye movement (Figure 2). After the last training the individual strategies used by the volunteers were assessed in a personal open interview. No strategy was advised to the volunteers at the beginning of the training, to enable the volunteers to develop their own strategy in interplay with the feedback, to develop the most efficient strategy.

#### 2.3.1. Passive Period

Within the passive period the volunteers fixated the central cross. During this period, the mean gamma (during gamma sessions) or alpha (during alpha sessions) current density power in the defined ROIs was computed and used as a relational index for the following feedback periods.

Anytime a blink or eye movement occurred during the passive period, the corresponding segment (1 s) was removed to assure an artifact free baseline measurement. In addition, the session was stopped if more than 20% of the passive period contained artifacts.

#### 2.3.2. Feedback Period

Before each session, volunteers were verbally informed about an upcoming gamma or alpha session. During the feedback periods, volunteers were instructed to increase the presented value, which expressed the percentage change to the passive baseline. The feedback value on screen was computed and refreshed with a time resolution of 1 s. We avoided a faster refresh of the feedback value and color of the bars, in order to avoid rapid perceptual changes that could manipulate neural activity. During the feedback period two bars monitored EOG (above bar) and EMG artifacts (below bar) occurring within the past second of feedback training. Thus, volunteers were informed about a successful increase of activity in the defined frequency range without an influence of artifacts if the value increased and the two bars turned green. Respectively, the bars turned red as EOG or EMG artifacts occurred, and the percent value was set to zero (Figure 2).

#### 2.3.3. Success Display

In order to keep the volunteers motivated, a “success display” was presented for 9 s after each feedback period. The success display informed the volunteers about a successful or rather unsuccessful feedback period. The position of the ball in the game layout changed based on the intentionally increased values during the passed feedback period without an influence of artifacts. Thus, only values that were successfully increased during artifact free segments were used for ball movement. High values resulted in large distance movements of the ball, whereas low values resulted in shorter distance movements after a less successful feedback period. Hence, the success display was integrated in the design to keep the volunteers engaged and motivated, as larger ball movements were accomplished after a successful feedback period. The volunteers were ambitious to reach the goal as fast as possible. Additionally, the success display served as a short break for the participants between each trial. Participants had eight feedback periods to reach the target. Once the participant accomplished the goal, the session ended with a congratulations message and a new session was started. If the participant did not accomplished the goal within the given feedback periods, then the session was stopped and a new session was started.

### 2.4. Offline Data Analysis

For the EEG offline analysis, data of all electrodes was first divided into passive and feedback periods. The first 1000 ms of both periods were removed in order to avoid effects evoked by the stimulus onset. Each passive and feedback period was then divided into equal size segments of one-second length. The data was preprocessed and controlled for artifacts as described for the online processing of data.

To evaluate the effects of alpha- and gamma-band training in the ROIs, we applied a LORETA transformation of all EEG channels for alpha- and gamma-filtered segments. Artifact free segments (EOG and EMG bars green) were extracted and the median (less sensitive to extremely distributed data values) percent change of gamma/alpha activity in the ROIs compared to baseline was derived. These segments were also controlled offline for artifacts that were possibly not detected online. We conducted a repeated measures analysis of variance (ANOVA with factors session and frequency band) to compare the gamma- and alpha-band activity change during the alpha- and gamma-feedback periods.

To calculate the topographical distribution of BCI training, the electric potential differences (time domain EEG) in each electrode between the feedback and passive periods were calculated for both gamma and alpha periods of the last training day. To estimate the three-dimensional distribution of electrical activity (current density) of gamma- and alpha-neurofeedback training the sLORETA transformation (the KEY Institute for Brain-Mind Research, Zurich, Switzerland; Pascual-Marqui, [36]) was applied to the subtracted electric potential difference. The standardized LORETA method was applied for the source estimation, since LORETA achieves low localization error, whereas sLORETA is more exact and achieves far more less localization error. The cortex has been modeled as a collection of volume elements (voxels) in the digitized atlas provided by the Brain Imaging Center, Montreal Neurological Institute (MNI, Montréal, Canada; [37]). More detailed information on sLORETA can be read in [36,37,38,39].

## 3. Results

### 3.1. Analysis of Gamma and Alpha Activity in the ROIs

Within the last training day, we tested whether volunteers were able to selectively increase alpha and gamma power in the defined area. The percent change of gamma- and alpha-band activity to baseline was analyzed in both alpha and gamma sessions.

**Figure 3:**
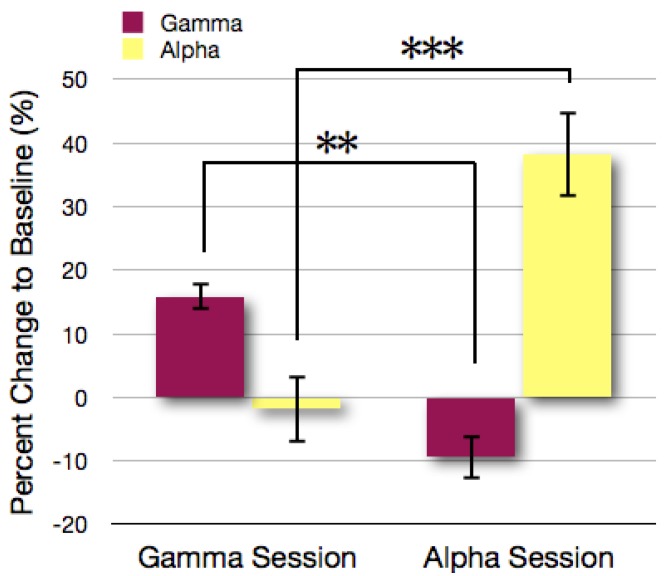
Percent change of gamma- and alpha-band activity in the gamma and alpha sessions within the last training day. Statistical tests revealed a higher increase of alpha power in the alpha sessions than in the gamma sessions [*t*(7) = 5.47, *p* < 0.001] and a higher increase of gamma power in the gamma sessions than in the alpha sessions [*t*(7) = 4.52, *p* < 0.01]. ** *p* < 0.01; *** *p* < 0.001.

Statistical tests revealed increased alpha power during the alpha sessions but not during the gamma sessions and increased gamma power within the gamma session but not during the alpha band related sessions (interaction of session (gamma/alpha) × frequency band (gamma/alpha) *F*(2,14) = 33,22, *p* < 0.001) (Figure 3). Further tests revealed that during the gamma band sessions, gamma band activity was significantly increased, while alpha band activity remained unchanged (*t*(7) = 3.16, *p* < 0.05). Respectively, this effect was reversed during the alpha band sessions, as alpha band activity was significantly increased and gamma band activity remained unaffected (*t*(7) = 4.9, *p* < 0.01). 

**Figure 4:**
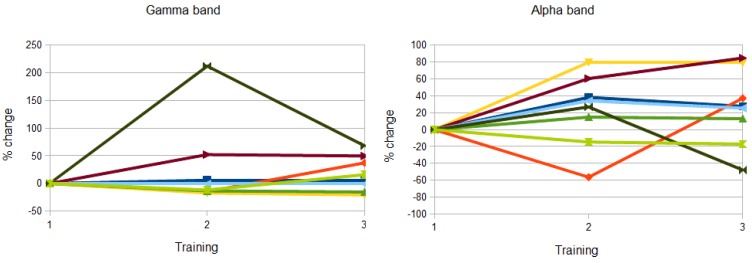
Training success for each volunteer as percent change to day one for the power values separate for each frequency band. For the gamma band, four subjects showed a positive effect, two showed a slight negative effect and two volunteers showed no training effect. For the alpha band, two different volunteers were not successful in enhancing the power across days.

Training success was analyzed by comparing the achieved gamma and alpha power values of the online estimation across training (ANOVA with repeated measures with factors frequency band and training). Results demonstrated that the volunteers learned to increase the power across days (training: *F*(1,7) = 6.5, *p* < 0.05), that the absolute power was larger in the alpha band (*F*(1,7) = 8.4, *p* < 0.05) and an identical learning across days (frequency band *x* training: *F*(2,14) = 0.5, n.s.). Individual data for each volunteer can be seen in Figure 4.

To further examine the performance of the volunteers the total amount of successful trials across trainings were analyzed (expressed as the percent change across days with day one as the reference). From day one to day two, a mean increase of 37% was observed (ranging from −29% to 96%) and from day one to day three, a mean increase of 80% was observed (−41% to 192%). Although three volunteers over all were not able to increase the total amount of trials, still the increase from training 2 to training 3 was significant (*t*(7) = 2.5, *p* < 0.05).

It should be noted that from the three volunteers that did not show an increase in the amount of trials, two volunteers showed a large increase in the amplitude parameter across training. This result may indicate a different strategy of the volunteers. Therefore, it can be concluded that the majority of volunteers (seven of eight) showed a clear training effect across the days.

The measured baseline values for gamma and alpha, in both gamma and alpha periods, did not show any significant differences (both n.s.), which reassured a common condition in both periods during baseline measurement.

### 3.2. Topographical Analysis of BCI Training

**Figure 5:**
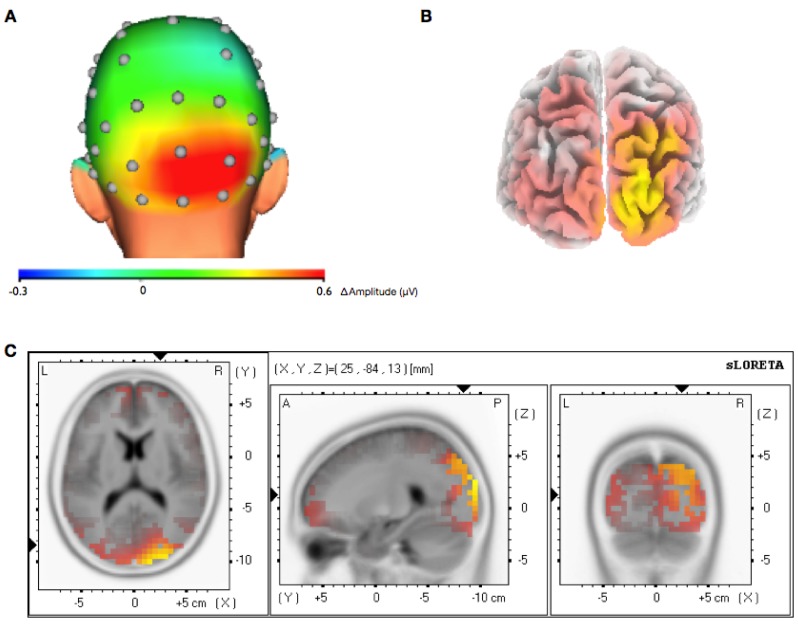
Topographic and spatial distribution of gamma band increase. (**A**) Topographic representation of the average change of the gamma-band activity (around 40 Hz) during the feedback period compared to the passive period within the last training session. The maximum change is localized at the occipital lobes close to the trained ROIs; (**B**) sLORETA analysis of the subtracted electrode potential difference of the gamma feedback sessions compared to the passive baseline. The yellow area represents the maximum estimated change of the gamma band activity to baseline. (*Back view*); (**C**) Horizontal, sagital and coronal view of the maximum change of the gamma-band activity.

**Figure 6:**
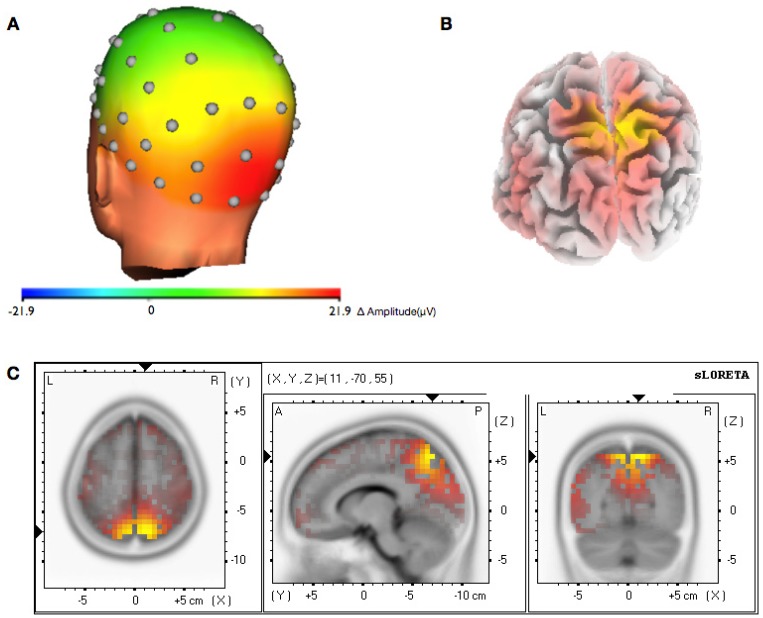
Topographic and spatial distribution of alpha band increase. (**A**) Topographic representation of the average change of the alpha-band activity (8–12Hz) during the feedback period compared to the passive period within the last training session. The maximum change is localized at the occipital and parietal lobes; (**B**) sLORETA analysis of the subtracted electrode potential difference of the alpha feedback sessions compared to the passive baseline. The yellow area represents the maximum estimated change of the alpha band activity to baseline. (*Back view*); (**C**) Horizontal, sagital and coronal view of the maximum change of the alpha-band activity.

The recording of the EEG channels over the whole scalp allowed the calculation of the topographic specificity of the feedback effect in both alpha and gamma sessions. This analysis revealed that the increase of the gamma band activity in the gamma sessions was limited to occipital electrodes and was not accompanied by a general increase over the whole scalp (Figure 5A). Results of the alpha sessions revealed a more widespread activation, as the alpha feedback training in the ROIs increased alpha in a wider range in the occipital lobe and also the parietal lobes (Figure 6A).

To estimate the three-dimensional distribution of electrical activity (current density) the gamma band/alpha band increase during the feedback periods was compared against baseline using sLORETA. The resulting images show the difference between gamma feedback periods and gamma baseline in the gamma sessions (Figure 5B,C) and the difference between alpha feedback periods and alpha baseline in the alpha sessions (Figure 6B,C). The maximum increase of gamma-band activity in the gamma sessions is located approximate to the trained ROIs and shows a focused distribution, while the maximum increase of alpha-band activity in the alpha sessions is located outside the trained ROIs and not that focused.

### 3.3. Analysis of Efficiency of Artifact Control

To assess the effect of artifact control during BCI training we conducted the amount of artifact contaminated alpha/gamma segments (at least one of the artifact bars red) within the first and last training days. Results revealed a significant decrease of artifact contaminated segments across training (reduction of 17% from first to last training day, *F*(1,7) = 6.02, *p* < 0.05). Thus, our results demonstrate the efficiency of artifact control during neurofeedback training as volunteers learned to control artifacts across training.

## 4. Discussion

In the present study, we designed an advanced BCI method to assist participants to switch between modulation of alpha- and gamma-band oscillations in the visual cortex. The BCI method used an online artifact control for artifact suppression, a special visual display design to avoid distraction and yet motivate volunteers and a source based BCI approach to limit the training to a distinct neural area. In particular, we trained both alpha and gamma frequency bands in an alternating sequence in the LOC and evaluated the effects of alpha- and gamma-band modulation on spatial and frequency specificity. Our results demonstrate an intentional influence on slow and fast oscillatory brain states by a source based BCI approach and the ability of the volunteers to deliberately switch between both states. Analyses of topographical characteristics revealed a gamma band increase that was restricted to the visual cortex with a maximum close to the ROIs and in comparison a more widespread alpha activity during alpha band increase. The advanced BCI approach offers a non-invasive method for a selective modulation of different ongoing oscillatory activity in selective brain regions.

Oscillatory brain activity in the gamma band is a rare applied frequency range in neurofeedback methods even though it is important for multiple cognitive processes [10,12,40]. In a recent study, initial attempts have been made to enhance gamma band activity in the occipital region [41]. The present approach used source localization for the gamma band training, included an online artifact control and compared the effects against alpha band modulations. 

With respect to our research question, we can state that (i) the online feedback of eye and muscle artifacts in addition to the feedback value clearly improved BCI training. Artifacts caused by EOG, microsaccades or EMG activity can results in undesired changes in the brain signals. For a BCI method based on the gamma-frequency band it is important to address the possible EMG artifacts. In particular, because EMG activity has a wide range, being maximal at frequencies higher than 30 Hz [42,43] and, accordingly, in a common range as the gamma-band activity. A recent discussion raised concerns regarding the neural origin of gamma-band activity [22], which provided evidence that increased gamma-band activity can be induced by microsaccades. Furthermore, EEG artifacts in general can invalidate the generation of the inverse solutions [29]. Hence, EOG, microsaccadic, and EMG artifacts were considered during BCI training and our results revealed a suppression of artifacts within the last training day compared to the first training day. Thus, our results clearly demonstrate that an additional feedback of artifacts during BCI experiments is essential and in fact assists the individual to learn to gain a better control of the actual physiological signals.

Artifacts, which occurred during BCI training, were visualized by two bars above and below the feedback value. The visual display was kept simple and the information was presented in the visual field in order to avoid eye movement or distraction. In the past, attempts have been made to report and to display artifact information during neurofeedback training [29]. However, in particular for the training of high frequency oscillations it is important to minimize perceptual change or the induction of eye movements that could affect the measured oscillatory activity. In the present approach, this was integrated by a color modification of the two bars that represented the source of artifacts (eye blinks, movements, microsaccades, and muscle contamination).

During the success display volunteers had a break, while they were informed about their performance in increasing oscillations in the past feedback period. Volunteers were motivated by the game display as successful feedback periods resulted in faster ball movements towards the goal. Thus, the visual display of our BCI approach included a simple and motivating display that avoided distraction during feedback sessions. In addition, this display allows the presentation of additional information (such as detailed artifact information) for a better preparation of the next feedback period.

With respect to our research question, (ii) the results show that all participants were able to learn a selective switch between modulations of alpha- and gamma-band activity. As individual strategies differed, most participants reported using a visual imagery strategy (visualizing a concrete figure, object or number at fixation) during the gamma periods and reported being relaxed during the alpha periods. The results of the training revealed that the majority of volunteers (seven of eight) were able to learn an influence over the used parameters, although the training was short. In many studies, more training is used to establish a stable influence over the brain activity and the individual data of our study suggested, that many volunteers could benefit from additional training. Although, all training data showed reliable effects across training over the whole group, some volunteers only showed an effect in the amount of successful trials, but without a strong effect on the amplitude of both frequency bands and some volunteers improved mainly the consistency of the influence. These results may indicate the differences in the strategies reported by the volunteers and the need for a longer training. Furthermore, it is known that even without neurofeedback training, the gamma-band activity increases when the subject is doing visual imagery and alpha-band activity increases during relaxed status. Therefore, the additional benefit of the neurofeedback approach should be tested against different cognitive training without feedback of brain signals. Analyses of the ROIs during the alpha and gamma periods revealed a clear increase of gamma activity during the gamma sessions and a clear increase of alpha activity during the alpha sessions. Thus, volunteers learned to increase activity in both alpha and gamma frequency bands in the predefined ROIs demonstrating that ongoing alpha and gamma band oscillations can be manipulated by a source based BCI approach and in particular in a specific brain region. The results further demonstrated the specificity of the modulations with respect to the frequency range. This specificity of the neurofeedback approach, with respect to a distinct frequency range and area, may be the benefit of the neurofeedback approach against more unspecific cognitive trainings.

With respect to our research question (iii) and (iv) results of sourced based BCI training with the LORETA method clearly demonstrated that participants learned to intentionally increase neural activity in the alpha and gamma band over the visual cortex. The topographical distribution of the estimated three-dimensional electrical activity of the gamma-band increase to baseline showed a selective enhanced effect in the visual cortex. The maximum effect was measured in the right lateral occipital lobe, close to the trained ROIs. Results of the topographical and spatial distribution of the alpha band increase demonstrated a rather widespread effect in the trained lateral occipital and in the occipito-parietal region, with a maximum effect in the superior parietal lobe. Thus, our results are in agreement with previous studies implicating the origin of posterior alpha rhythm from occipito-parietal areas, where it is modulated by visual input [44,45,46]. Our results demonstrate that a selective modulation of fast neural activity in a predefined neural region with a non-invasive BCI approach is feasible (v). Although, the manipulation of the alpha-band frequencies induced an increase in our specified ROI, the maximum increase was localized in the superior parietal lobe.

As a consequence for future research, when using a source based approach to modulate different frequencies in a specified ROI one has to account for a possible source of this effect outside the selected area and an offline source analysis is necessary to allow conclusions about a functional relation between modulations of the state of a distinct neural area and information processing. In a previous study, LORETA based BCI was used to train individuals to enhance low beta (16–20 Hz) and to suppress low alpha (8–10 Hz) in the anterior cingulate cortex (ACC) [29]. Based on this study, a further study explored the effect of training in the ACC on anterior regions [47].

In summary, we developed an advanced BCI approach with artifact control to selectively increase oscillations in different frequencies in a predefined region in the brain. We demonstrated the effectiveness of artifact control during BCI training, as volunteers learned to decrease artifacts across training. We showed that volunteers learned to selectively increase both alpha- and gamma-band oscillations in the LOC with a source based BCI approach. The topographical distribution of gamma- and alpha-band training revealed a restricted increase of gamma-band activity close to the specified ROIs and a more widespread increase of alpha-band activity. In a recent study we applied the developed BCI method to train participants in the alpha- and gamma-band range in order to directly assess the behavioral consequences [21]. Therefore, we used the developed BCI modules as a reactive BCI to adaptively present visual object stimuli within well-described states of oscillatory activity.

Visual objects presented in states of increased gamma band activity showed a processing advantage compared to low levels of gamma-band activity or increased alpha-band oscillations. Thus, with the developed BCI method we were able to provide further evidence for the specific functional role of prestimulus gamma-band oscillations for visual object processing.

In conclusion, the selective manipulation of ongoing oscillatory activity in specific brain regions underlines the value of the advanced BCI approach as a method for the examination of a more direct relationship between oscillatory brain states and behavior.
